# Comparison of the penetration depth in mouse brain *in vivo* through 3PF imaging using AIE nanoparticle labeling and THG imaging within the 1700 nm window†

**DOI:** 10.1039/d3na00871a

**Published:** 2023-12-06

**Authors:** Yingxian Zhang, Jincheng Zhong, Hui Cheng, Jie Huang, Zhenhui Li, Chi Zhang, Zhiang Gao, Zhourui Xu, Gaixia Xu, Ping Qiu, Ke Wang

**Affiliations:** a Key Laboratory of Optoelectronic Devices and Systems of Ministry of Education and Guangdong Province, College of Physics and Optoelectronic Engineering, Shenzhen University Shenzhen 518060 China pingqiu@szu.edu.cn kewangfs@szu.edu.cn; b School of Biomedical Engineering, Shenzhen University Medical School, Shenzhen University Shenzhen Guangdong 518055 China

## Abstract

3-Photon microscopy (3PM) excited at the 1700 nm window features a smaller tissue attenuation and hence a larger penetration depth in brain imaging compared with other excitation wavelengths *in vivo*. While the comparison of the penetration depth quantified by effective attenuation length *l*_e_ with other excitation wavelengths have been extensively investigated, comparison within the 1700 nm window has never been demonstrated. This is mainly due to the lack of a proper excitation laser source and characterization of the *in vivo* emission properties of fluorescent labels within this window. Herein, we demonstrate detailed measurements and comparison of *l*_e_ through the 3-photon imaging of the mouse brain *in vivo*, at different excitation wavelengths (1600 nm, 1700 nm, and 1800 nm). 3PF imaging and *in vivo* spectrum measurements were performed using AIE nanoparticle labeling. Our results show that *l*_e_ derived from both 3PF imaging and THG imaging is the largest at 1700 nm, indicating that it enables the deepest penetration in brain imaging *in vivo*.

## Abstract 2

Herein, we systematically studied 3PF imaging and THG imaging in living mice at different excitation wavelengths within the 1700 nm window, thus exploring the optimal wavelength for the excitation of MPM within the 1700 nm window.

## Graphical abstract

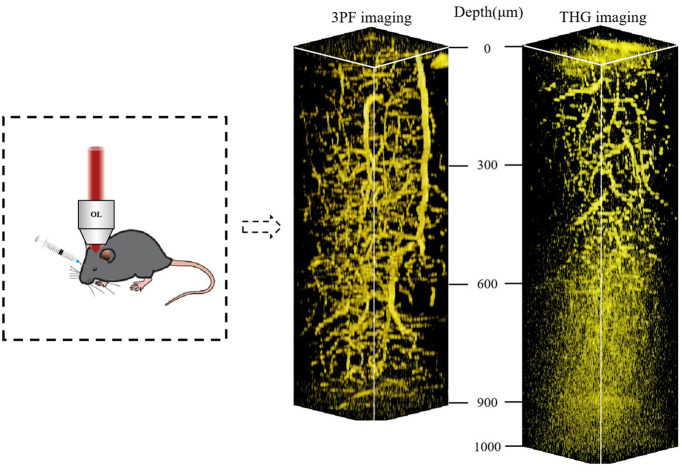

## Introduction

The visualization of brain structures is one of the essential stages in the study of brain science. Optical imaging has been widely adopted for both structural and functional brain deciphering.^1^ Rapidly developing and advancing optical imaging technologies are contributing to the development of brain and neuroscience research. Among them, multiphoton microscopy (MPM) is a nonlinear optical technology^2–6^ with significant advantages, such as non-invasiveness, high-spatial resolution, and deep penetration.^1,7–10^ Depth enhancement is the goal of any imaging technique. Towards this goal, different MPM technologies have been developed: (1) higher order nonlinear three-photon imaging^7,11^ can be used to suppress the surface background^1,3^ and (2) shifting to longer excitation wavelengths to reduce tissue attenuation and hence increase the multiphoton signal level in deep tissue.^1,7,8,12–15^

To reduce the excitation light attenuation caused by absorption and scattering, the excitation wavelengths are commonly selected within the following four “tissue optical windows”:^2,16–18^ NIR-I (800 nm window, 650–950 nm),^16,19^ NIR-II (1300 nm window, 1100–1350 nm),^17,20,21^ NIR-III (1700 nm window, 1600–1840 nm),^1,17,20^ and NIR-IV (2200 nm window, 2100–2300 nm).^7,8,17^ These four optical windows have been confirmed by ex vivo transmittance measurement, tissue phantom simulation, and *in vivo* imaging.^7,8,11,17^ MPM at different excitation wavelength windows were compared, and it was found that MPM with excitation at the 1700 nm window enables the largest imaging depth *in vivo*.^22^

The attenuation of the excitation light was determined by the combined effect of absorption and scattering. Effective attenuation length (*l*_e_) is a quantitative measure of the maximum achievable imaging depth, so we quantitatively compared the attenuation of the excitation light to the imaging depth by *l*_e_.^8,12,23^*l*_e_ is defined as *l*_e_ = *l*_a_*l*_s_/(*l*_a_ + *l*_s_),^11^ where *l*_a_ is the absorption length and *l*_s_ is the scattering length. A larger *l*_e_ means less excitation light attenuation (in the tissue) and the ability to penetrate deeper into the tissue. Experimentally, Kobat *et al.* compared two-photon fluorescence microscopy (TPM) at 775 nm and 1280 nm, and the imaging depth at 1280 nm excitation was twice as deep as that with 775 nm excitation due to a larger *l*_e_.^13^ Wang *et al.* compared *l*_e_ at different excitation wavelengths of 1300 nm, 1450 nm, 1500 nm, 1550 nm, and 1700 nm, and found that *l*_e_ at 1300 nm and 1700 nm were larger, which verified that the 1300 nm and 1700 nm excitation windows are suitable for deeper tissue imaging.^12^ Besides, *l*_e_ at 1700 nm is larger than that at 1300 nm. Chen *et al.* showed *in vivo* 3PM imaging with 2200 nm excitation and compared it with 1700 nm excitation, which verified that the *l*_e_ at 2200 nm excitation is smaller than at 1700 nm excitation.^8^ All the above results point to the 1700 nm window as the deepest penetration window for deep tissue imaging. However, there has been no comparison of *l*_e_ within this broad window (as broad as 240 nm), leaving the open question as to which wavelength within this window is better.

To explore the optimal wavelength for the excitation of MPM within the 1700 nm window, in this study, we systematically investigated 3-photon imaging in living mice at 3 different excitation wavelengths, *i.e.*, 1600, 1700, and 1800 nm within this window. For a fair comparison, exogenous fluorophores with the same 3PF emission spectrum excited at these 3 wavelengths were used. Recently, Deng *et al.* developed nanoparticles with aggregation-induced emission (AIE) properties, *i.e.*, MTTCM NPs. MTTCM NPs have good biocompatibility and optical properties and can generate strong 3PF under NIR-III excitation.^24^ Third-harmonic generation (THG) imaging was also performed, the results of which corroborate with those of 3PF imaging. By using *l*_e_ to quantitatively compare the attenuation of the excitation light and the imaging depth, our results indicate that the 1700 nm excitation wavelength penetrates the deepest within the 1700 nm window.

## Materials and methods

### Experimental setup

**Fig. 1:**
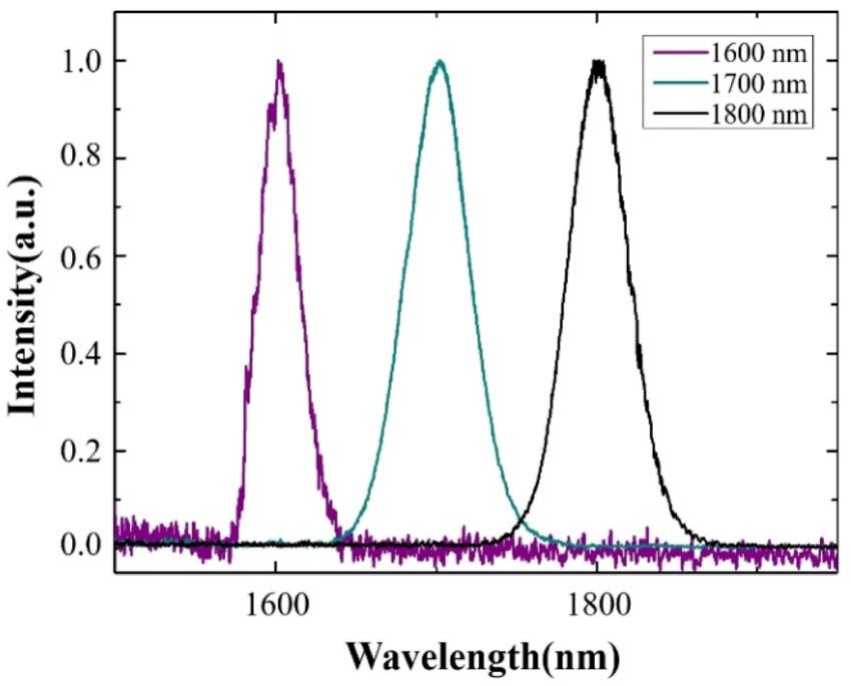
Measured spectrum for 1600 nm, 1700 nm, and 1800 nm soliton pulses.

Our experimental system is shown in Fig. S1 of ESI.† Based on the nonlinear optical effect of the soliton self-frequency shift (SSFS), we used a high-energy 1550 nm, 6 MHz femtosecond pulses laser (FLCPA-02CSZU, Calmar Laser) as a pump source for a 3 m large mode field (LAM) fiber (LMA-PM-35, NKT Photonics) to generate femtosecond soliton pulses at 1600, 1700, and 1800 nm. The output light pulses were filtered using a 1575 nm long-pass filter (1575LP, Omega Optical), 1650 nm long-pass filter (Yi Zhao Photonics Technology), and 1725 nm long-pass filter (1725LP, Omega Optical) to obtain the 1600 nm, 1700 nm, and 1800 nm laser sources required for imaging, respectively. The spectra of 1600 nm, 1700 nm, and 1800 nm soliton pulses were measured using an optical spectrum analyzer (OSA203B, Thorlabs), as shown in Fig. 1.

The filtered excitation light passed through a beam expander, consisting of a scan lens (LA5763-D, Thorlabs) and a tube lens (ACA254-200-D, Thorlabs) in a multiphoton microscope (MOM, Sutter) and then focused onto the sample through a water immersion objective (XPLN25XSVMP2, Olympus) with a working distance of 2 mm and NA = 1.05 for *in vivo* brain imaging. Both, the generated fluorescence and THG signals were detected using a GaAsP photomultiplier tube (H7422p-50, Hamamatsu). The maximum optical powers after the objective lens were 21 mW for 1600 nm, 29 mW for 1700 nm, and 36 mW for 1800 nm. For 3PF imaging of MTTCM NPs, a 633 nm long-pass filter (BLP01-633R-25, Semrock) was used to block the THG signal generated by red blood cells. A 535/50 nm bandpass filter (ET535/50-2p-18deg, Chroma), 560/94 nm bandpass filter (FF01-560/94-25, Semrock), and 605/70 nm bandpass filter (ET605/70M-2P, Chroma) were used to acquire THG signals generated by 1600 nm, 1700 nm, and 1800 nm excitation, respectively. All the images were acquired at a speed of 2 s per frame, with a pixel size of 512 × 512.

### Animal procedures

All animal procedures were performed in accordance with the Guidelines for Care and Use of Laboratory Animals of Shenzhen University and approved by the Animal Ethics Committee of Shenzhen University Medical School (Approval No.: IACUC-202300036). All mice were obtained from the Guangdong Medical Laboratory Animal Center, China. The experiments were performed using adult female mice (C57BL/6J, 8-10 weeks old) for imaging. Mice were anesthetized using a gas anesthesia system (Matrx VIP 3000, Midmark) and isoflurane. The body temperature of the animals was maintained at 36.5 °C using a heating pad and injected with a 50 μL h^−1^ dose of 5% dextrose. A 3 mm diameter craniotomy centered at 2 mm posterior and lateral to the Bregma point was performed. A home-made metal piece was tightly glued to the skull using dental cement and a coverslip (5 mm diameter) was used to seal the cranial window. Prior to imaging, cerebral vessels were labeled by orbital injection of 100 μL of MTTCM NPs.

## Results and discussion

### *In vivo* characterization of THG and 3PF emission spectra in circulating blood

**Fig. 2:**
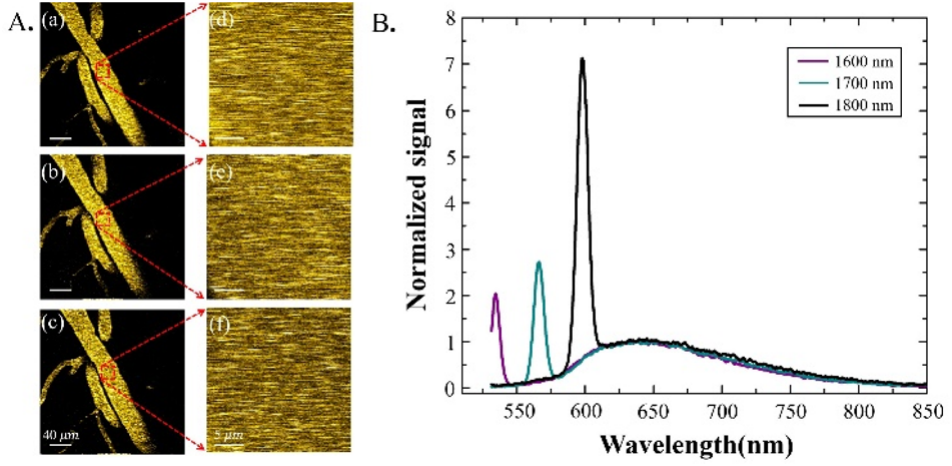
3PF imaging-guided 3PF emission spectra measurement of MTTCM NPs in the mouse brain *in vivo*. (A) 3PF images of MTTCM NPs-labeled blood vessels excited at 1600 nm (a and d), 1700 nm (b and e), and 1800 nm (c and f). (B) Correspondingly measured 3PF emission spectrum of MTTCM NPs.

In order to quantitatively compare the attenuation of the excitation at different wavelengths, the effects of the emission wavelength have to be characterized and considered. In order to do this, we performed emission spectra measurements guided by 3-photon imaging in the mouse brain *in vivo*. The same blood vessel 40 μm below the brain surface (Fig. 2A(a–f)) was selected for imaging and spectral measurements. The measured 3-photon emission spectra of MTTCM NPs in the circulating blood under 1600 nm, 1700 nm, and 1800 nm excitation are shown in Fig. 2B.

The measured results show two distinct features: (1) the 3PF emission spectra of MTTCM NPs under three different excitation wavelengths were the same, which is a verification not only of Kasha's rule in the 3PF regime but also of the fact that potential difference in the emission wavelength under different excitation wavelengths is eliminated. (2) THG peaks on the left side of the emission spectra are clearly visible, which is due to the flowing red blood vessels inside the vessel.^25^ For a fair comparison, THG signals *in vivo* have to be removed, which was accomplished by the 633 nm long-pass filter, yielding pure 3PF signals for subsequent comparisons.

### *In vivo* 3PF imaging and *l*_e_ measurements

**Fig. 3:**
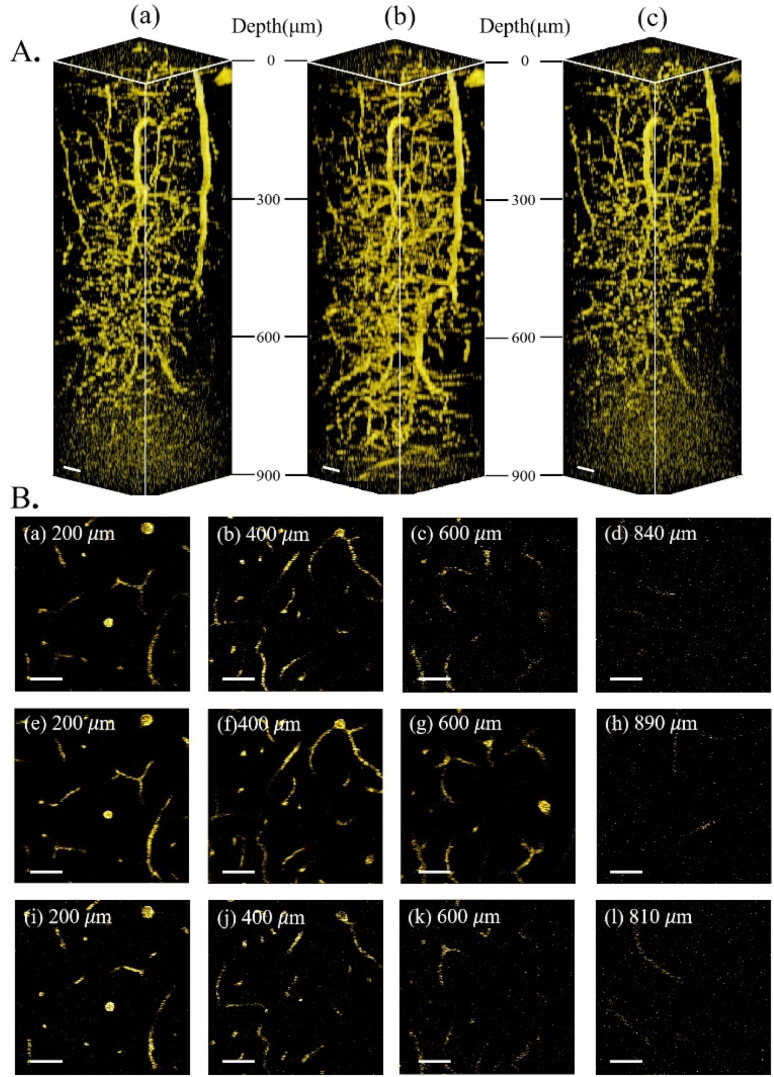
3PF imaging of the mouse brain *in vivo*. (A) 3D reconstruction of 3PF images in the mouse brain excited at 1600 nm (a), 1700 nm (b) and 1800 nm (c). (B) 2D images from A at different imaging depths below the brain surface (indicated in each figure) excited at 1600 nm (a–d), 1700 nm (e–h), and 1800 nm (i–l). Scale bars: 50 μm.

Having characterized and isolated the 3PF signals, 3PF imaging was performed in the same mouse brain within the same region. Excited at 1600 nm, 1700 nm, and 1800 nm, we acquired 3D reconstructions of 3PF images to a depth of 900 μm below the brain surface (Fig. 3A). The information and experimental parameters related to the penetration depth of different excitation wavelengths within the 1700 nm window (in this work and previous studies) are shown in Table S1 of ESI.† Based on the 3PF imaging results, we next measured *l*_e_ to determine the maximum imaging depth achievable with different wavelength excitations. Imaging at the same brain region with the same 3PF emission spectra, the difference in *l*_e_ only stems from different excitation wavelengths.

**Fig. 4:**
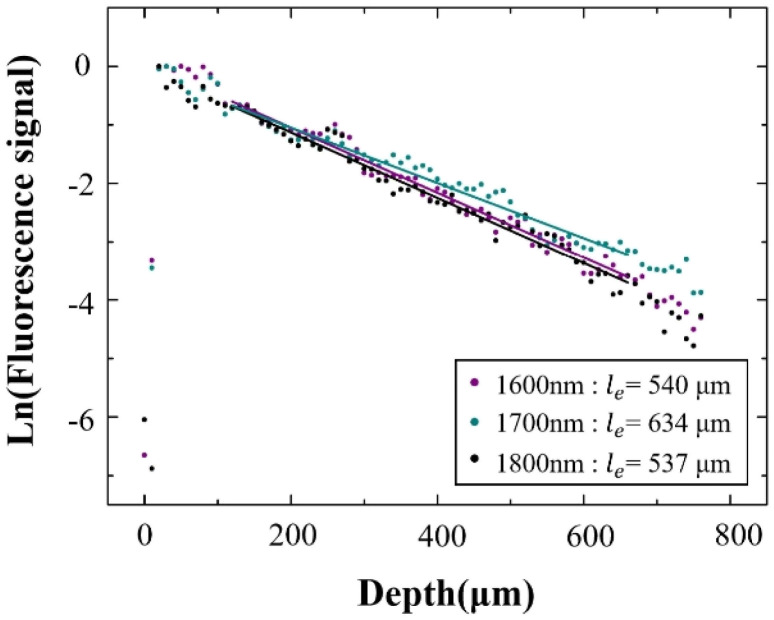
Normalized 3PF signal decay curve excited at 1600 nm, 1700 nm, and 1800 nm. The dots are the measured data, and the lines are linear fits to the measured data.

The *l*_e_ was calculated from the slope of the fit in Fig. 4. Since the 3PF signal is proportional to the cube power, we have 
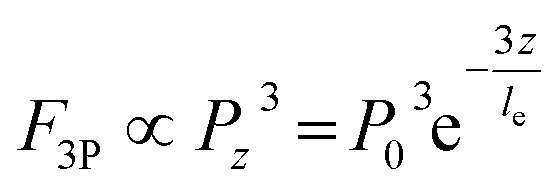
,^12^ so *l*_e_ is given by *l*_e_ = 3/slope. In accordance with a previous reference, we chose the average of the brightest 0.1% pixels at each depth in the *x*–*y* images as the fluorescence signal.^13^ Following this procedure, *l*_e(3PF)_ (1600 nm) = 540 μm, *l*_e(3PF)_ (1700 nm) = 634 μm, and *l*_e(3PF)_ (1800 nm) = 537 μm. Therefore, our results showed that *l*_e(3PF)_ (1700 nm) > *l*_e(3PF)_ (1600 nm) > *l*_e(3PF)_ (1800 nm), which indicates that 1700 nm excitation attenuates least upon propagation in the brain *in vivo*, due to the combining effect of brain absorption and scattering.

### *In vivo* THG imaging and *l*_e_ measurements

**Fig. 5:**
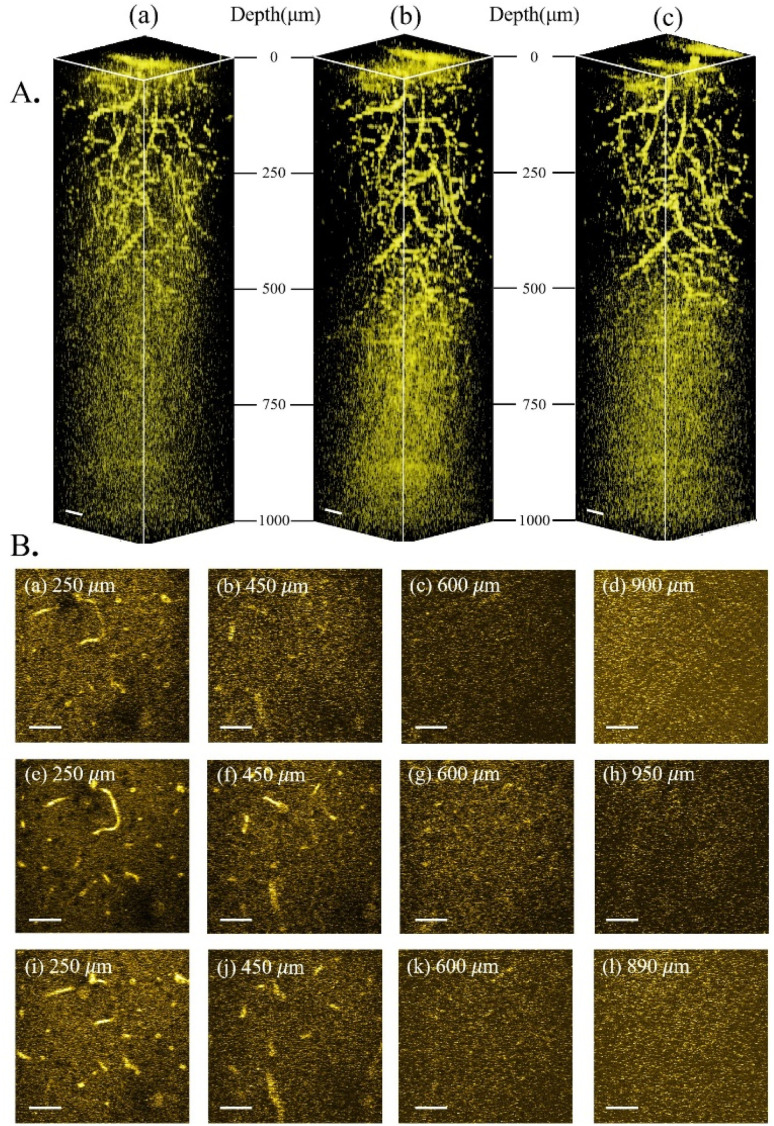
THG imaging of the mouse brain *in vivo*. (A) 3D reconstruction of THG images excited at 1600 nm (a), 1700 nm (b), and 1800 nm(c). (B) 2D THG images from A at different imaging depths below the brain surface (indicated in each figure) excited at 1600 nm (a–d), 1700 nm (e–h), and 1800 nm (i–l). Scale bars: 50 μm.

**Fig. 6:**
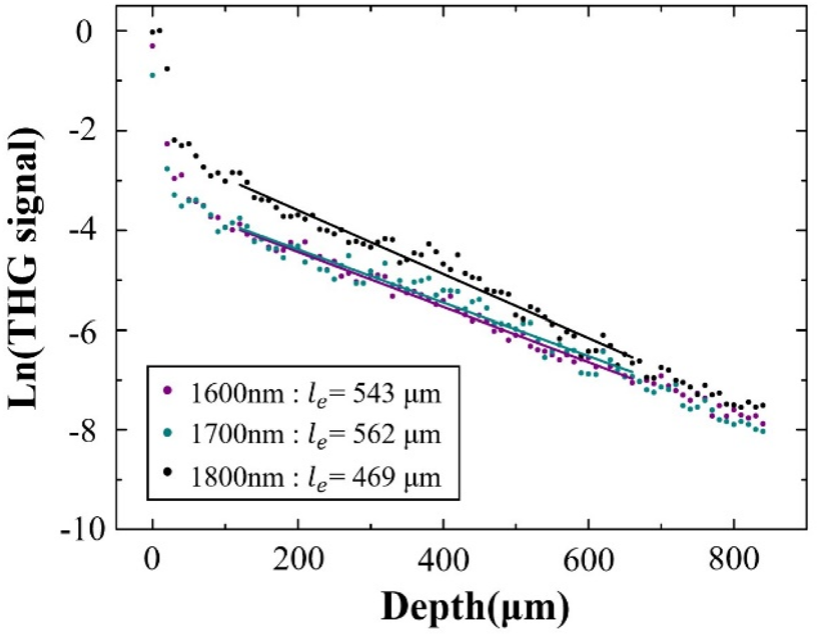
Normalized THG signal decay curve excited at 1600 nm, 1700 nm, and 1800 nm. The dots are the measured data, and the lines are linear fits to the measured data.

As further experimental verification of the above experimental conclusion from 3PF imaging, we next imaged the same region of the same mouse brain with THG under 1600 nm, 1700 nm, and 1800 nm excitation, and calculated *l*_e_ from THG images. No AIE nanoparticles were injected for THG imaging. The reconstructed THG 3D stacks are shown in Fig. 5A. Blood vessels are also visible from the THG imaging due to the presence of red blood vessels. Following the same procedure, *l*_e_ was calculated from the fitted slopes in Fig. 6 from THG imaging for all three excitation wavelengths, yielding *l*_e(THG)_ (1600 nm) = 543 μm, *l*_e(THG)_ (1700 nm) = 562 μm, and *l*_e(THG)_ (1800 nm) = 469 μm, respectively. Thus, we reach the same conclusion that *l*_e(THG)_ (1700 nm) > *l*_e(THG)_ (1600 nm) > *l*_e(THG)_ (1800 nm), agreeing with that revealed by 3PF imaging.

## Conclusions

In order to investigate the wavelength-dependent excitation decay in the mouse brain *in vivo* at the 1700 nm window, in this study, we demonstrate analysis based on 3PF imaging enabled by biocompatible AIE nanoparticles, and THG imaging in mouse brain *in vivo*, excited at 1600 nm, 1700 nm, and 1800 nm within the 1700 nm window. *In vivo* 3PF spectral measurements enable clear distinction and filtering of pure 3PF signals from THG signals *in vivo*. Derivation of the effective attenuation length *l*_e_ from both 3PF and THG imaging *in vivo* points to 1700 nm as the least attenuating excitation wavelength for *in vivo* brain imaging. Thus, we conclude that MPM excited at 1700 nm is the most suitable for deep brain imaging within the 1700 nm window, in terms of the excitation decay.

## Author contributions

K. W. and Y. Z. conceived and designed the experiments. Y. Z., H. C., J. H. and Z. L. conducted the experiments. Z. X. and G. X. performed the experiments on synthesis. Y. Z., J. Z., C. Z., Z. G. analyzed the results. Y. Z. wrote the manuscript. P. Q. and K. W. modified the manuscript and supervised the entire project. All authors have approved the final version of the manuscript.

## Conflicts of interest

There are no conflicts to declare.

## Supplementary Material

NA-006-D3NA00871A-s001
