# Supplementary material for: Comparison of the penetration depth in mouse brain in vivo through 3PF imaging using AIE nanoparticle labeling and THG imaging within the 1700 nm window
Source: Nanoscale Adv. 2023 Dec 6;6(2):511–5. doi: 10.1039/d3na00871a (PMC10790977; doi:10.1039/d3na00871a)
Supplement: NA-006-D3NA00871A-s001 [file NA-006-D3NA00871A-s001.pdf]

Electronic Supplementary Material (ESI) for Nanoscale Advances.
This journal is © The Royal Society of Chemistry 2023

Supporting Information

Comparison of penetration depth in mouse brain in vivo through 3PF imaging labelled by AIE nano particles and THG imaging within the 1700 nm window

Yingxian Zhang,[a] Jincheng Zhong,[a] Hui Cheng,[a] Jie Huang,[a] Zhenhui Li,[a] Chi Zhang,[a] Zhiang Gao,[a] Zhourui Xu,[b] Gaixia Xu,[b] Ping Qiu *[a] and Ke Wang *[a]

[a] Key Laboratory of Optoelectronic Devices and Systems of Ministry of Education and Guangdong Province, College of Physics and Optoelectronic Engineering, Shenzhen University, Shenzhen 518060, China.

[b] School of Biomedical Engineering, Shenzhen University Medical School, Shenzhen University, Shenzhen, Guangdong, 518055, China.

E-mail: pingqiu@szu.edu.cn, kewangfs@szu.edu.cn

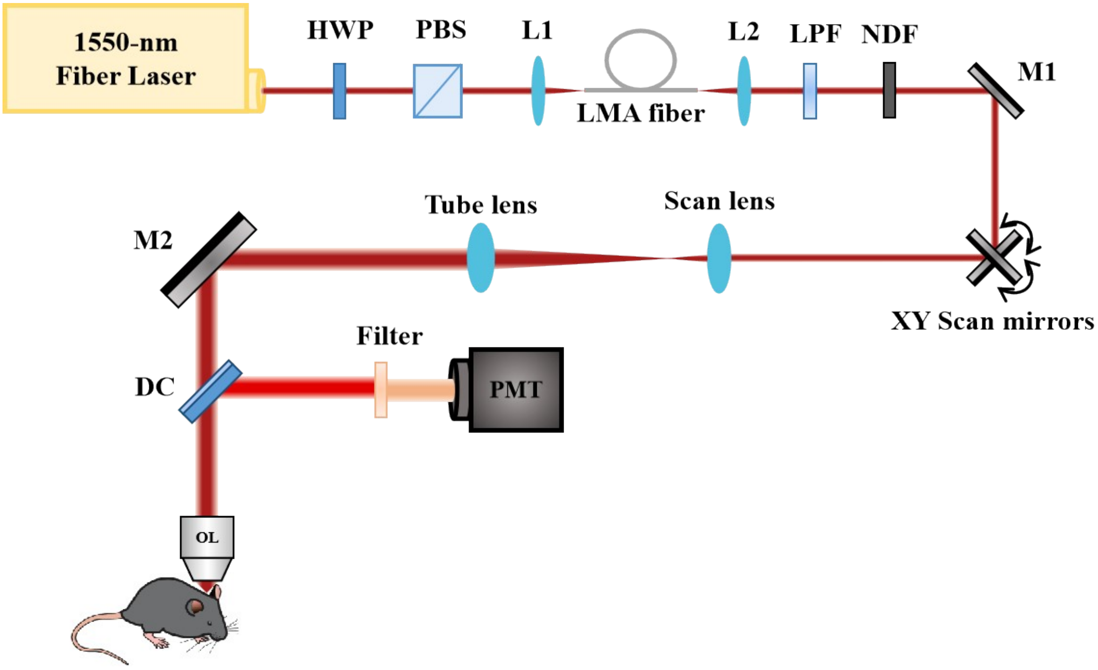

Fig. S1 The experimental setup of 3PM imaging system. HWP: half-wave plate; PBS: polarization beam splitter; L1 and L2: focusing and collimating lens; LPF: long-pass filter; NDF: neutral density filter; M1 and M2: silver-coated mirror; DC: dichroic mirror; Filter: band-pass filter; PMT: GaAsP photo-multiplier tube; OL: objective lens.

Table S1 In vivo mouse deep-brain imaging of different fluorescence indicator with 1700nm window

Wavelength (nm)	Materials	MPM	Concentration	Depth (μm)	Repetition rate (MHz)	Ref.
1600	MTTCM NPs	3PM	1 mM	840	6	*
1610	BONAPs	3PM	50 mg/kg	1680	1	[1]
1617	ICG	2PM	2 mM	2000	1	[2]
1620	SR101	3PM	3.3 mg/ml	1340	1	[3]
1660	MTTCM NPs	3PM	2 mM	1900	1	[4]
1665	Texas red	3PM	700 μM	1650	1	[5]
1665	Qtracker655	3PM	2 μM	2100	1	[6]
1665	DPNA-NZ	3PM	2 mM	1700	1	[7]
1665	DPCZ-BT	3PM	2 mM	1860	1	[8]
1700	DCTBT	2PM	2 mM	2180	1	[9]
1700	MTTCM NPs	3PM	1 mM	890	6	*
1720	OEFT NPs	3PM	550 μM	1696	1	[10]
1800	MTTCM NPs	3PM	1 mM	810	6	*

* Experimental parameters from this article

Note: The repetition rate of the laser used in this experiment was 6 MHz, while all other experiments used a repetition rate of 1 MHz. Because of the higher repetition rate used in this experiment, the pulse energy is lower and the imaging depth is smaller.

References

1. M. X. Liu, B. B. Gu, W. B. Wu, Y. K. Duan, H. J. Liu, X. Q. Deng, M. Z. Fan, X. M. Wang, X. B. Wei, K. T. Yong, K. Wang, G. X. Xu and B. Liu, *Chem. Mat.*, 2020, **32**, 6437-6443.
2. H. Cheng, S. Tong, X. Q. Deng, H. J. Liu, Y. Du, C. He, P. Qiu and K. Wang, *Optics Letters*, 2019, **44**, 4432-4435.
3. H. J. Liu, J. Q. Wang, Z. W. Zhuang, J. X. He, W. H. Wen, P. Qiu and K. Wang, *Journal of Biophotonics*, 2019, **12**, 10.
4. X. Q. Deng, Z. R. Xu, Z. J. Zhang, W. J. Zhang, J. A. Li, L. Zheng, X. L. Chen, Y. Pan, P. Qiu, D. Wang, G. Xu and K. Wang, *Advanced Functional Materials*, 2022, **32**, 2205151.
5. W. H. Wen, Y. X. Wang, H. J. Liu, K. Wang, P. Qiu and K. Wang, *Journal of Biophotonics*, 2018, **11**, 6.
6. H. Liu, X. Deng, S. Tong, C. He, H. Cheng, Z. Zhuang, M. Gan, J. Li, W. Xie, P. Qiu and K. Wang, *Nano Letters*, 2019, **19**, 5260-5265.
7. Z. R. Xu, Z. J. Zhang, X. Q. Deng, J. A. Li, Y. H. Jiang, W. C. Law, C. B. Yang, W. J. Zhang, X. L. Chen, K. Wang, D. Wang and G. X. Xu, *Acs Nano*, 2022, **16**, 6712-6724.
8. J. A. Li, Z. J. Zhang, X. Q. Deng, Z. R. Xu, L. Wang, G. X. Xu, K. Wang, D. Wang and B. Z. Tang, *Biomaterials*, 2022, **287**, 11.
9. D. Li, X. Q. Deng, Z. R. Xu, D. L. Wang, G. X. Xu, P. Y. Zhang, P. Qiu, W. X. Xie, D. Wang, B. Z. Tang and K. Wang, *Advanced Functional Materials*, 2023, **33**, 2303967.
10. S. L. Li, X. Q. Deng, H. Cheng, X. Z. Li, Y. P. Wan, C. Cao, J. Yu, Y. Liu, Y. Yuan, K. Wang and C. S. Lee, *Acs Nano*, 2022, **16**, 12480-12487.
